# Evolution of phenotypic disparity in the plant kingdom

**DOI:** 10.1038/s41477-023-01513-x

**Published:** 2023-09-04

**Authors:** James W. Clark, Alexander J. Hetherington, Jennifer L. Morris, Silvia Pressel, Jeffrey G. Duckett, Mark N. Puttick, Harald Schneider, Paul Kenrick, Charles H. Wellman, Philip C. J. Donoghue

**Affiliations:** 1 Bristol Palaeobiology Group, School of Earth Sciences, University of Bristol, Bristol, UK; 2 School of Biological Sciences, University of Bristol, Bristol, UK; 3 Milner Centre for Evolution, Department of Life Sciences, University of Bath, Bath, UK; 4 Institute of Molecular Plant Sciences, School of Biological Sciences, University of Edinburgh, Edinburgh, UK; 5 The Natural History Museum, London, UK; 6 Center of Integrative Conservation, Xishuangbanna Tropical Botanical Garden, Chinese Academy of Sciences, Menglun, China; 7 School of Biosciences, University of Sheffield, Sheffield, UK

**Keywords:** Evolution, Plant evolution, Natural variation in plants

## Abstract

The plant kingdom exhibits diverse bodyplans, from single-celled algae to complex multicellular land plants, but it is unclear how this phenotypic disparity was achieved. Here we show that the living divisions comprise discrete clusters within morphospace, separated largely by reproductive innovations, the extinction of evolutionary intermediates and lineage-specific evolution. Phenotypic complexity correlates not with disparity but with ploidy history, reflecting the role of genome duplication in plant macroevolution. Overall, the plant kingdom exhibits a pattern of episodically increasing disparity throughout its evolutionary history that mirrors the evolutionary floras and reflects ecological expansion facilitated by reproductive innovations. This pattern also parallels that seen in the animal and fungal kingdoms, suggesting a general pattern for the evolution of multicellular bodyplans.

## Abstract (web summary)

Clark and colleagues show that plant anatomical diversity evolved episodically over geologic time, reflecting ecological expansion facilitated by reproductive innovations. This is paralleled in animals and fungi, suggesting a general evolutionary mode for multicellular bodyplans.

## Main

Biological diversity is not continuously variable but rather is composed of clusters of self-similar organisms that share a common bodyplan. Systematists have long exploited these discontinuities in the structure of biological diversity as a basis for imposing taxonomic order. However, the discontinuous nature of organismal design is intrinsically interesting, alternatively interpreted to reflect constraints in the nature of the evolutionary process, adaptive peaks or contingencies in evolutionary history. Much empirical work has shown that phenotypic diversity (disparity) is distributed unequally among lineages and across time, with many clades achieving maximal disparity early in their evolutionary history limited subsequently to expanding the range of variation within these early limits^1,2^. However, these observations have been based largely on studies of animal clades and it is unclear whether they are more generally applicable. Analyses of plant phenotypic disparity have focused on single groups of characters such as branching architecture^3,4^, reproductive organs^5–9^, leaf architecture or shape^10,11^ and vasculature^12^, and have been restricted to subclades or individual lineages^13–15^. Here we attempt an integrated characterization of the evolution of phenotypic disparity in the plant kingdom with the aim of testing the generality of macroevolutionary patterns observed in the animal kingdom.

## Results

**Extended Data Fig. 1:**
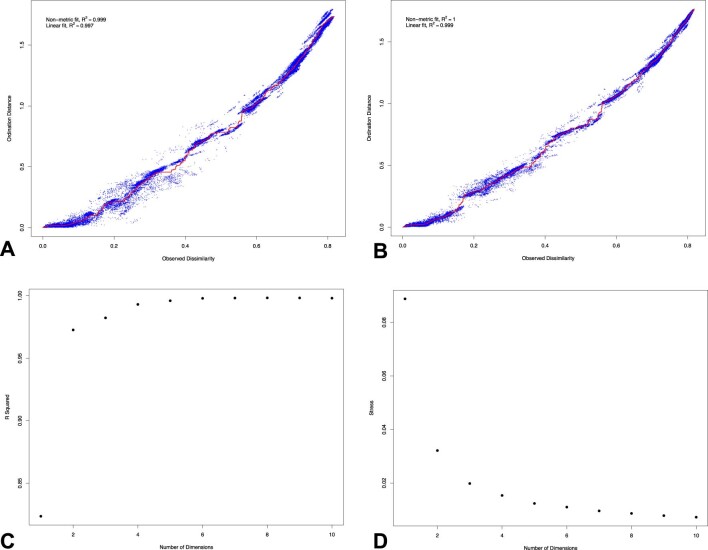
Stress indicated by an increasing number of axes in the NMDS analyses. Stressplots indicate the goodness of fit for NMDS analyses with 2 dimensions **(a)** and 3 dimensions **(b)**. The goodness of fit increases with more dimensions **(c)** while the stress decreases **(d)**.

We compiled a phenotype dataset from published character matrices^16–22^, revising and expanding character and taxon sampling to encompass all aspects of morphology, from sperm cell structure to gross plant architecture, and the breadth of Kingdom Viridiplantae. The resulting supermatrix is composed of 548 traits for 248 living taxa representing every phylum, amounting to 131,280 data points (data available online). The vast diversity of angiosperms makes proportional sampling difficult, although our sampling approximately reflects known species diversity (Spearman’s *⍴* = 0.83, *P* = 0.01). The ensuing dataset was ordinated using non-metric multidimensional scaling (NMDS) which summarizes variance onto a prescribed number of axes; sensitivity tests confirmed that the variation in the dataset can be represented effectively on two axes (Extended Data Fig. 1). By definition, NMDS ordinations are non-metric but parallel analyses of the same dataset using principal coordinate analysis (PCoA; which generates ordinations with metric axes) show that pairwise distances derived from NMDS and PCoA analyses are highly correlated (Mantel test, *r* = 0.99, *P* < 0.001; Extended Data Fig. 1) and so we interpret the NMDS as approximately metric.

**Fig. 1:**
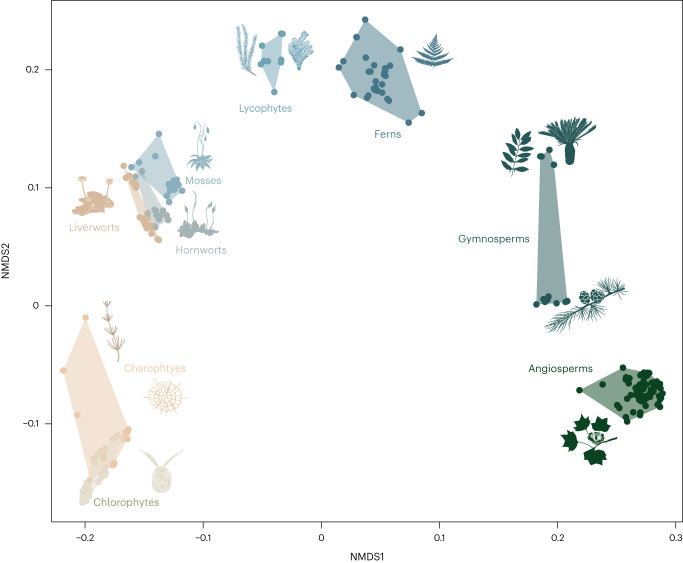
Empirical morphospace of the plant kingdom. A morphospace derived from all 548 characters for 248 extant taxa. The axes summarize morphological disparity derived from the observed dissimilarity between living taxa (calculated using Gower’s index) subjected to NMDS. A convex hull was fitted around each major lineage.

**Extended Data Fig. 2:**
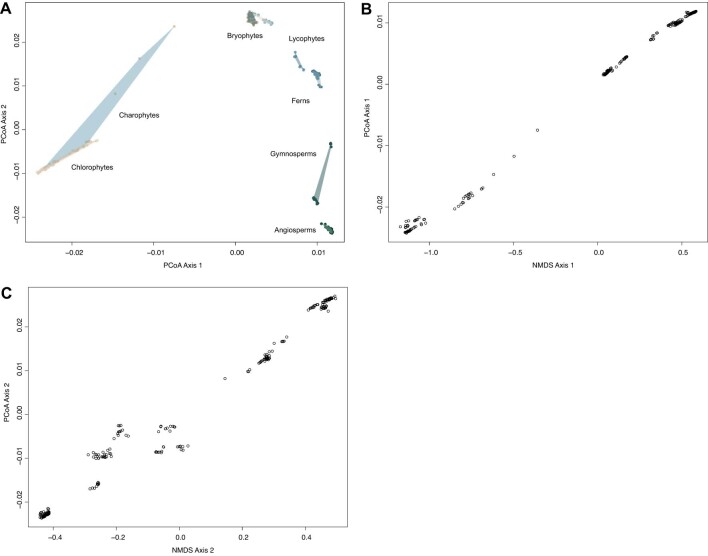
A metric morphospace constructed using Principal Coordinates Analysis (PCoA) of the distance matrix. A metric morphospace constructed using Principal Coordinates Analysis (PCoA) of the distance matrix **(a)**. The correlations between scores of the PCoA and NMDS analyses for the first axis **(b)** and second axis **(c)** are shown.

The resulting ordination shows that the greatest dissimilarity between groups separates land plants (Embryophyta) from green algae, vascular and non-vascular plants, and seed plants from spore-bearing plants (Fig. 1). The main clades comprise discrete clusters within morphospace (bar green algae, which are paraphyletic^23^), separated by large unoccupied regions, arranged along NMDS axis 1 (NMDS1). The conspicuous arch in the ordination is reminiscent of the ‘arch’ or ‘horseshoe’ artefact, but in this instance it reflects shared characters among clades at the opposite ends of NMDS1, viz. seed plants and charophyte algae (for example, loss of flagellated sperm and complex gametophytes). Removal of these characters from the dataset yields a more linear distribution of taxon clusters within morphospace (Extended Data Fig. 2).

**Extended Data Fig. 3:**
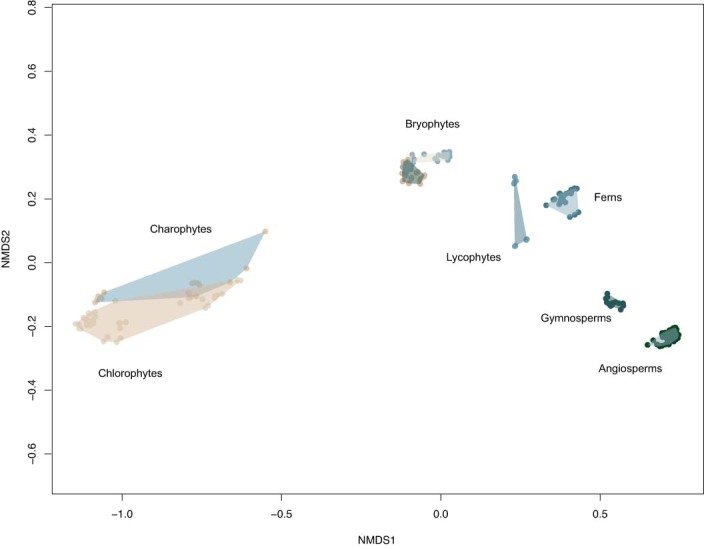
A morphospace of extant species after the removal of characters related to sperm and mobile zoospores. Ordination of the dataset after removal of these characters leads to reduced variance on NMDS2.

Charophyte algae show the highest mean disparity (Extended Data Fig. 3), although this is a paraphyletic grouping and much of the observed disparity is accounted for by the difference between the multicellular and unicellular taxa that comprise the charophyte grade (Fig. 1). Gymnosperms exhibit a broad range of variation comprising two widely separated clusters composed of Ginkgoopsida + Cycadopsida and Pinopsida, reflecting the large differences in their organization. The comparatively low disparity among angiosperms is perhaps surprising because, superficially, much of the phenotypic disparity is attributed to floral characteristics. We have sampled clades at equivalent taxonomic rank and, for all their diversity, the reproductive and life history traits of the angiosperms are conserved^24^. Sampling proportional to diversity would doubtless increase angiosperm disparity; however, our analyses show that disparity is not correlated with species diversity (Spearman’s *⍴* = 0.23, *P* = 0.55), although certain clades show low levels of both disparity and diversity; for example, hornworts^25^. There is no correlation between clade age and mean disparity (Pearson’s *r* = 0.541, *P* = 0.16), although the phylogenetic (patristic) distance between taxa is correlated with their morphological distance (Mantel test, *r* = 0.3, *P* = 0.001). Despite this, there are instances where convergence is more apparent than conservatism, such as the position of the charophyte alga *Chara* relative to the embryophytes (Fig. 1).

### Disparity of life cycles, vegetative and reproductive traits

**Fig. 2:**
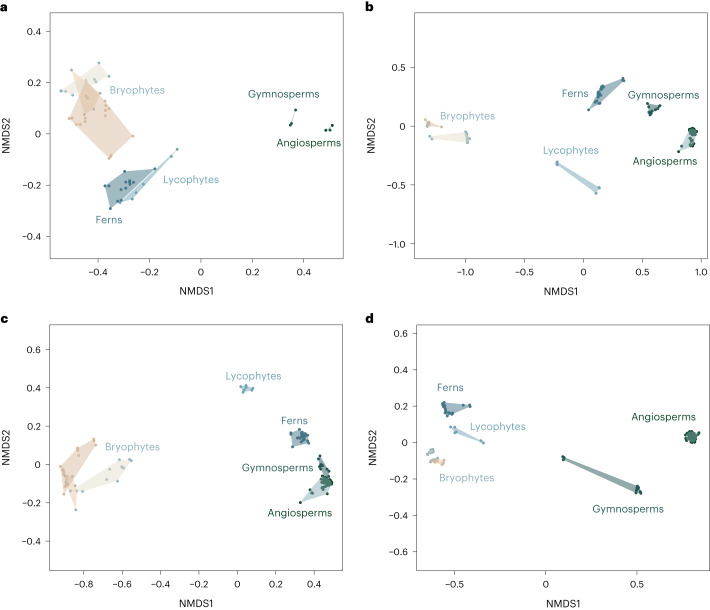
Morphospaces constructed from characters reflecting different growth modes and life cycles. **a**, The gametophyte (haploid) generation. **b**, The sporophyte (diploid) generation. **c**, Vegetative structures. **d**, Reproductive structures.

Dividing our dataset into different suites of characters produced morphospaces with contrasting patterns (Fig. 2). We initially divided the morphospace to reflect the two life cycles of land plants (Fig. 2a,b) because alternation of multicellular diploid and haploid phases is a defining trait of land plants^26^. Bryophytes exhibit the most disparate gametophytes and the least disparate sporophytes. Conversely, the highly reduced gametophytes of seed plants result in their occupation of the smallest (although highly distinct) regions of morphospace. Lycophytes and ferns show broad morphospace occupation in both generations; their sporophytes are closest to seed plants, whereas their gametophytes are closer to the bryophytes.

Morphospaces built around vegetative characters (stem anatomy, branching and appendages) exhibit less phylogenetic structure; there is greater convergence between lineages and divergent evolution within lineages (Fig. 2c). This is seen most clearly in the extent of overlap on NMDS1 of ferns, gymnosperms and angiosperms (Fig. 2c). Reproductive characters reinforce the distances between lineages (Fig. 2d), which are most exaggerated in pollen, spore and embryological characters.

### Disparity and complexity

**Fig. 3:**
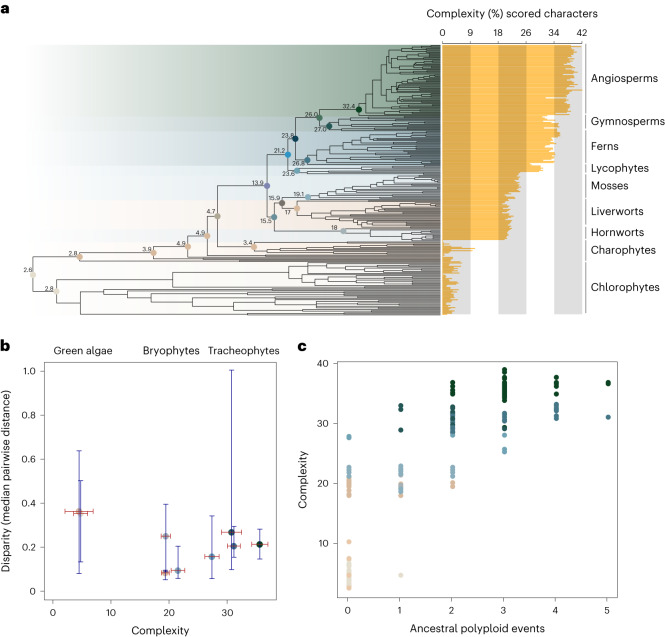
Evolution of phenotypic complexity within the plant kingdom. **a**, The distribution and reconstruction of complexity across the plant kingdom. The scale on the *x* axis reflects the number of positively coded characters for each taxon included in the analysis. The numbers associated with nodes on the tree reflect estimates for the complexity of these ancestral nodes. **b**, Relationship between phenotypic disparity (median pairwise distance) and phenotypic complexity, for each of the plant phyla. A significant phylogenetically corrected correlation was observed (Pearson’s *r* = 0.76, *P* < 0.001). **c**, Relationship between phenotypic complexity and the number of ancestral polyploidy events that lineage has experienced.

The concepts of phenotypic disparity and complexity are often used interchangeably although they have distinct meanings. Disparity is a property of groups, describing their phenotypic differentiation, whereas complexity is a property of individuals, describing the number of part types and their differentiation^27–29^. To characterize the evolution of plant phenotypic complexity we recoded our matrix to capture the number of characters that are coded present in each extant species, facilitating comparison across clades and over phylogeny. The results show that complexity is lowest in unicellular algae (Fig. 3a). Among the chlorophyte and charophyte algae, Zygnematophyceae, the sister lineage to the land plants, are among the least complex; this finding is of note because it indicates a marked decrease in complexity from the shared ancestor of Zygnematophyceae and Embryophyta (Fig. 3a). By contrast, there is a step-change in complexity associated with the origin of land plants, with successive innovations associated with land plant clades reflected in more muted increases in complexity. The origin of land plants is associated with a jump in values, from 4.7 in the crown-anhydrophyte to 13.9 in the crown-embryophyte; all living land plants have values over 18. Among bryophytes, liverworts exhibit some of the lowest values of phenotypic complexity of all land plants, which is unsurprising because they have long been considered among the simplest of land plants, serving as an experimental proxy for the ancestral embryophyte (for example^30^), or having lost phenotypic characters that the other bryophyte lineages have retained^23,31^. However, reductions in liverwort complexity have occurred in their recent evolutionary history. The origin of tracheophytes (vascular plants) is associated with the next most notable increase in complexity, reflecting the many phenotypic novelties associated with their bodyplan, including vascular tissues, axial branching and true roots. Euphyllophytes are generally more complex than their lycophyte relatives, and many gymnosperms are comparable with their monilophyte and angiosperm relatives, the latter resolved as the most complex of all members of the plant kingdom. We found no clear relationship between disparity and complexity (Fig. 3b), with some highly disparate groups composed of taxa exhibiting low complexity (charophyte algae) and lineages with low disparity composed of taxa characterized by moderate levels of complexity (bryophytes). Thus, phenotypic complexity does not appear to be a prerequisite for disparity in the plant kingdom. We observed a significant phylogenetically corrected correlation between species complexity and the number of polyploidy events in their evolutionary history (*r* = 0.76, *P* < 0.001; Fig. 3c), with the mean complexity values increasing progressively from species with no history of genome duplication to those that have four or five polyploidy events in their evolutionary history. This corroborates the widely held view that whole-genome duplication is a mechanism by which complexity has evolved within the plant kingdom^32^.

### Evolution of phenotypic disparity

**Fig. 4:**
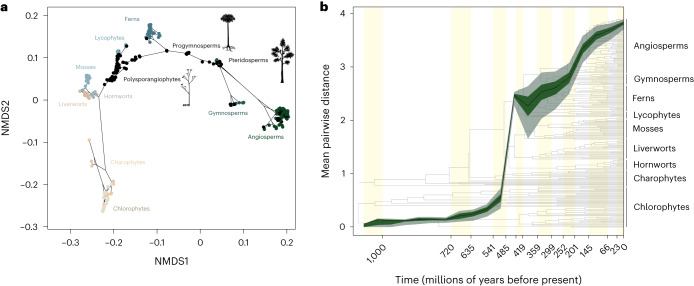
Fossil morphospace and disparity through time. **a**, Empirical phylomorphospace of the plant kingdom including the same sample of extant taxa plus 160 fossil taxa, and their inferred ancestors. The axes summarize morphological disparity derived from the observed dissimilarity between taxa (calculated using Gower’s index) subjected to NMDS. A convex hull was fitted around each major lineage. Fossil taxa are shown as black dots. The tree represents a summary of the current hypotheses of phylogenetic relationships, and the character states at each node were estimated through stochastic character mapping across a sample of trees. **b**, Cumulative phenotypic disparity (sum of variances) through time, estimated using a time-calibrated phylogeny including fossil taxa whose phylogenetic position could be reliably estimated. The solid line represents the median from 1,000 bootstrap replicates, with the shaded regions representing the 2.5% and 97.5%, and 25% and 75% percentiles. Species divergence dates were obtained from Morris et al.^56^. Disparity through time was estimated using dispRity^54^ in R, under a model of gradual evolution with time bins every 50 million years. Orange bands represent geological periods.

Our characterization of plant phenotypic disparity is a census of modern diversity. To infer its evolutionary history, we constructed a morphospace by reconstructing ancestral states based on the traits of the extant species, their phylogenetic relationships and a model of stochastic character evolution. The phylomorphospace (Fig. 4a) reflects the relationship between phylogeny and morphospace occupation, showing that some currently unoccupied regions of morphospace were once occupied by phylogenetic intermediates of the living clades. However, this analysis reflects only a net perspective on the evolution of plant phenotypic disparity; approaching the true pattern requires the inclusion of fossil taxa that have left no direct extant descendants. Indeed, the fossil record preserves unique character combinations not seen in extant plants^33^ and so fossils have the potential to change radically a perception based on living clades alone.

**Extended Data Fig. 4:**
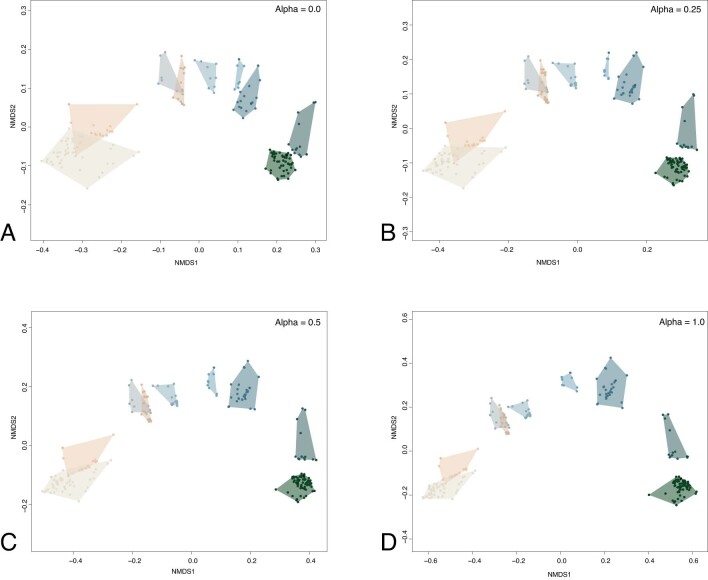
Morphospaces based on pairwise distance metrics developed to address the hierarchical characters among characters. The alpha parameter affects the influence of contingent characters by weighting primary characters according to the number of contingencies, from no influence (alpha = 0) to greater influence (alpha = 1). Morphospaces constructed from different alpha values are shown for alpha = 0 **(a)**, alpha = 0.25 **(b)**, alpha = 0.5 **(c)** and alpha = 1 **(d)**.

To this end, we introduced 160 fossil taxa including eophytes^34^, other stem-tracheophytes, zosterophylls, lycopsids, progymnosperms and pteridosperms, as well as fossils that are assigned to the major extant lineages. Plant macrofossils are rarely informative about the entire plant phenotype, resulting in large proportions of non-random missing data. Dissimilarity indices, such as Gower’s coefficient, can accommodate missing data to some degree^35^ but ordination of our raw data led to fossil taxa clustering together based on shared missing data (Extended Data Fig. 4). This can be overcome through probabilistic phylogenetic inference of missing character states^36^ based on their phylogenetic relationships to better-known living relatives (Methods).

The phylomorphospace constructed from both fossil and extant taxa shows that fossil taxa do not alter the fundamental pattern of morphospace occupation seen in extant taxa and none lie beyond the regions of morphospace occupation circumscribed by the extant phylomorphospace (Fig. 4a). Nevertheless, fossil taxa expand upon the range exhibited by the extant clades alone, effectively corroborating the prediction of morphospace occupation by the extant phylomorphospace. Eophytes, polysporangiophytes and zosterophylls populate the stem-tracheophyte, stem-lycophyte and stem-euphyllophyte branches of the phylomorphospace, whereas progymnosperms and pteridosperms populate the stem- and early crown-spermatophyte branches. The morphological distances between algae and land plants, and between angiosperms and gymnosperms in particular, are maintained even with the inclusion of fossils.

### Disparity through time

The hypothesis of maximal initial disparity predicts that clades establish the limits of morphospace occupation early in their evolutionary history and that subsequent diversity is largely confined to these early limits^1,2^. In most tracheophyte lineages, fossil taxa are located at or close to the limits of extant phenotypic disparity (Fig. 4a), compatible with a pattern of high early disparity within these lineages, followed by a plateau where morphospace is packed rather than expanded^37^. However, this pattern does not hold for the plant kingdom as a whole, which exhibits a pattern of episodically increasing phenotypic disparity. This begins with a period of low variance associated with the chlorophyte and charophyte algae, followed by a rapid increase through the late Cambrian to Silurian associated with the colonization of land by plants and the establishment of the major land plant lineages (Fig. 4b). Subsequently, disparity increases at a slower rate through the middle and late Palaeozoic to the early Mesozoic, followed by a sharp increase during the Triassic that reflects the diversification of gymnosperms and ferns and the origin of angiosperms. Finally, disparity increases at a low rate from the Cretaceous to the present.

## Discussion

This is the first comprehensive analysis of plant phenotypic disparity and its evolution, encompassing living and fossil diversity. Our analysis shows that clades are not equal in their phenotypic variation and there is no clear relationship between diversity and disparity. Plant morphospace is not evenly occupied, with the living clades comprising discrete clusters, leaving large areas of morphospace unoccupied. The distinctiveness of clades is driven largely by reproductive characters, whereas vegetative characters exhibit convergence on morphospace occupation by phylogenetically distinct lineages. The relative dominance of life cycles among bryophytes and tracheophytes impacts on their disparity, with non-seed plants exhibiting the greatest disparity for gametophyte characters, whereas bryophytes exhibit little disparity for sporophyte characters.

In part, the clumpy nature of plant morphospace occupation is a result of the extinction of phylogenetic intermediates that once bridged clade-based clusters, as evidenced by our phylomorphospace analysis and the inclusion of fossil species. In effect, extant plant lineages have contracted from areas occupied by their forebears. However, the clustered occupation of morphospace also results from the divergence of these clades within morphospace, from their shared ancestors and from one another. Fossil taxa populate many of the branches on the phylogeny within morphospace, but some branches remain conspicuously depauperate, including stem-angiosperms, stem-conifers and stem-embryophytes (fossil species are known that might occupy some of these branches, but there are few credible candidates for the embryophyte stem). Overall, the phylomorphospace demonstrates exploration of new regions of morphospace throughout the evolutionary history of plants. This is seen at the level of all characters but is mostly strongly associated with the evolution of reproductive novelties, such as those associated with the origin of embryophytes, seed plants and angiosperms, but also with realization of the ecological opportunities that those reproductive novelties afforded.

This broad pattern is compatible with previous characterizations of plant disparity that have focused on individual organ systems, such as leaves^10^ and reproductive structures^8^, both of which show evidence for the exploration of morphospace through the evolutionary history of euphyllophytes and tracheophytes, respectively. Oyston et al.^13^ undertook a comprehensive characterization of plant phenotype, but focused on the evolution of individual clades (leptosporangiate ferns, conifers, pines, palms, water lilies, as well as angiosperms more generally), most of which exhibit a rapid initial increase in variation that is subsequently maintained. This view is compatible with our results wherein the extant variation of many clades falls largely within the bounds established by extinct relatives (for example, lycophytes within polysporangiophytes and spermatophytes generally). Logically, given the sequential appearance of higher taxa, this cannot scale into a self-similar pattern for the kingdom as a whole^36^ and that is what our results show.

Our analysis of disparity through time bears out a pattern of episodically increasing disparity for the plant kingdom. The sharp increases in disparity that occur in the early Palaeozoic and mid Mesozoic coincide broadly with the transitions between the recognized three or four major evolutionary floras—early tracheophytes, Devonian seedless plants, Mesozoic gymnosperms and early seed plants, and the rise of angiosperms during the Jurassic/Cretaceous^38^—which have been associated with a succession of evolutionary novelties, viz. vascular tissue, true leaves, the seed and the flower, respectively.

Whole-genome duplication has often been invoked as a causal factor in plant macroevolution and, indeed, palaeopolyploidy has been associated with some of the lineages that exhibit the greatest expansions in morphospace occupation, such as spermatophyte and angiosperm stems^14^. Although comparable expansions are also associated with the embryophyte and tracheophyte stems, on which no ploidy events have been inferred, these branches are associated with pulses in gene family innovation^31,39^ that, arguably, have much the same effect in creating redundant genes available for neofunctionalization or the rewiring of gene regulatory networks.

Although some of the major plant clades exhibit patterns compatible with it, maximal initial disparity can be rejected, unquestionably, for the plant kingdom as a whole. Rather, plant phylogeny is characterized by episodically increasing variance associated with both reproductive and vegetative innovations (Fig. 2a,b) that have facilitated the invasion of stressful environments and ecological expansion^40^. Recent comparative genomic studies have shown that many key phenotypic novelties evolved long after the genes implicated in their development^39,41^. Thus, the episodic increases in plant disparity may have resulted from the realization of genomic and developmental potential through ecological opportunity. Valentine et al.^40^ argued that plants and animals exhibit different evolutionary dynamics as a consequence of (1) plants, but not animals, having continuously invaded stressful environments, and (2) plants having simple bodyplans and indeterminate growth compared with animals’ structurally complex bodyplans and determine growth; these are interpreted collectively to explain the fundamental differences in the timing of origin of major clades in the two kingdoms. To be sure, there are fundamental differences in the timing of origin of higher taxa in plants and animals^40^, but it is not clear whether this reflects differences in taxonomic practice versus evolutionary mode. The phenomenon of maximal initial disparity was grounded in analyses of animal clades^1,2^, although this may merely characterize the evolution of component clades, as in plants. Indeed, at the kingdom scale, both animals^36^ and fungi^42^ exhibit a pattern of episodically increasing phenotypic disparity. Analysis of the animal kingdom suggests that early burst patterns may characterize the evolution of fossilizable characters, rather than phenotypic characters more generally^36^. Furthermore, as in plants, major post-Cambrian expansions in animal morphospace occupation are associated with ecological transitions, including terrestrialization and flight^36^. Combined, these analyses of the major multicellular kingdoms suggest that a pattern of episodically increasing variance may be a general pattern for the evolution of multicellular bodyplans.

The canonical model of maximal initial disparity has been marshalled in support of evolutionary non-uniformitarianism^43^, reflecting greater evolvability early in the evolution of lineages, the capacity for fundamental innovation diminishing over time with, for example, the complexification of gene networks that regulate development^44^. Our analyses and others call into question the generality of the model of maximal initial disparity and, along with it, the idea that lineages lose their capacity for fundamental innovation over evolutionary time. At the kingdom level it appears that constraints can be overcome through the evolution of major innovations that have led to the occupation of whole new regions of morphospace. Experiments have revealed that mutations can produce phenotypes consistent with some of the major transitions in land plants, including inducing multicellularity in green algae or branching in bryophyte sporophytes^45,46^. These experiments provide a means of understanding how some land plant lineages could have escaped developmental constraints. However, the pattern of episodically increasing variance that we recover for the plant, animal and fungal kingdoms is compatible with a model in which evolutionary novelties vary in their capacity for innovation sensu^47^. Few component clades exhibit structure within phylomorphospace because of widespread convergence, whereas there is little or no overlap between these clades (clumpiness). This suggests that most evolutionary novelties are of small effect, whereas only a few led to fundamental innovation manifest as major expansions in morphospace occupation. Ecological challenge and opportunity appear to underpin innovation in all three multicellular kingdoms, realizing the potential of existing genomic and developmental novelties.

## Methods

### Matrix assembly

An initial character matrix was assembled to span the Viridiplantae (Chlorophyta + Streptophyta) by fusing the character matrices from cladistic studies of green algae, charophytes and bryophytes^16,17,48^, early tracheophytes and lycophytes^21^, ferns^18^, seed plants and gymnosperms^22^ and early angiosperms^19^. The characters represented all areas of plant morphology (cellular, developmental, vegetative and reproductive) and tissue types (sporophytic and gametophytic). Overlapping characters were reconciled between matrices to avoid repetition and the number of character states expanded to capture morphology across a greater number of clades. Additional taxa and characters were added to the matrix and in many cases the scoring of taxa was revised in light of more recent understanding of homology or re-examination of taxa (data available online).

### Inference of missing data

The distances between taxa were being poorly represented because of the non-random distribution of missing data in fossil taxa. We performed phylogenetic reconstruction under a Bayesian framework using the Mk model, in which the positions of extant taxa were constrained based on evidence from molecular systematics^23^, but the placement of fossil taxa was unconstrained. We ran 4 parallel chains for 10 million generations each and selected 100 random trees from the posterior distribution (data available online). Along each tree, we simulated the possible tip states using stochastic character mapping^49,50^. Stochastic character mapping calculates the conditional likelihood of each character state at each node in the tree, stochastically assigns node states based on their probability and then simulates character history along each branch. We fixed known tip states with a probability of 1, and for unknown and missing tip states allowed each possible tip state an equal prior probability. We ran 1,000 simulations per character per tree, and for each selected the state that had been sampled most frequently. We then estimated the most probable tip state and node state across all 100 trees to create a focal matrix which formed the basis for subsequent analyses.

### Constructing the Viridiplantae morphospace

**Extended Data Fig. 5:**
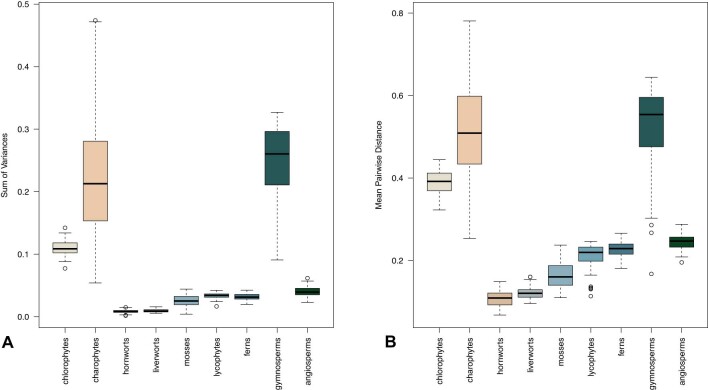
A comparison of disparity between lineages based on A. sum of variances and B. mean pairwise distance. Disparity is calculated as the mean pairwise distance between taxa within each lineage. The partial disparity represents the contribution of each lineage to the total morphological variation, calculated as the mean distance to the overall centroid. The bounds represent the maxima and minima estimated from 1000 bootstrap replicates of the data, with the boxes corresponding to the 25^th^ and 75^th^ percentile and the centre the median.

All ordination analyses were performed on the focal matrix. The distances between taxa were calculated using Gower’s similarity metric^51^, which treats all character states as unordered and can tolerate missing data from the matrix. In addition, Gower’s index does not count matching zeros in the calculation of dissimilarity, and so shared inapplicable characters do not contribute to the distance between taxa or their position within the morphospace^35^. The distance matrix was subjected to a NMDS multivariate analysis, with the number of dimensions constrained to 2. A stressplot was used to assess how well the data were represented in two dimensions and reported a strong relationship between the observed dissimilarity and the ordination distances (stress = 0.031, *r*^2^ = 0.99; Extended Data Fig. 5). Non-metric methods are better suited for ordinations with a large proportion of absences and non-ordered multistate characters but produce a morphospace that can be challenging to interpret, because the resulting space is non-Euclidean and the distances between taxa are non-metric. We repeated the analysis using metric methods, using the Euclidean distance between taxa and a PCoA, to test whether the NMDS analysis approximated a metric morphospace (Extended Data Fig. 1). The morphospace was constructed initially with only extant taxa, and then with the inclusion of fossil taxa.

A consensus phylogeny based on molecular evidence and our current understanding of the placement of fossil taxa was used to construct a phylomorphospace^19,21,22,50^. The position of the nodes within the morphospace was based on the distance between nodes and living taxa combined in a single ordination. Convex hulls were fitted around each major lineage to illustrate the occupied envelope of morphospace using vegan^52^.

### Disparity between lineages

Indices of disparity were calculated from the distance matrix. The disparity within lineages (mean disparity) was calculated as the mean pairwise distance between each taxon within the lineage. The partial disparity represents the contribution to the total morphological diversity of the kingdom and is calculated as the mean distance to the overall centroid for each taxon within a subclade, divided by *n* − 1, where *n* is the total number of taxa included in the analysis^53^. All calculations were performed on a sample of 1,000 bootstrap replicates of the distance matrix and were performed using the dispRity^54^.

### Disparity through time

Calculation of disparity through time was performed using the time-slicing approach^55^. We used a time-calibrated phylogeny containing 40 fossil taxa whose phylogenetic position could be robustly inferred (data available online). Analyses were based on the dissimilarity matrix and included the reconstructed ancestral node states for the phylogeny. We ran both punctuated and gradual models of evolution, with the punctuated model randomly selecting both accelerated and decelerated transformations. The matrix was bootstrapped 1,000 times to estimate the standard error at each time point.

### Dividing the morphospace

Characters within the matrix were subdivided into eight broad and non-mutually exclusive categories: sporophytic (250 characters), gametophytic (56 characters), branching and appendages (55 characters), shoot anatomy (45 characters), roots and symbionts (20 characters), zoospores and spermatozoids (97 characters), spores, pollen and embryology (93 characters), and sporophylls and sporangia (58 characters). We recalculated a distance matrix for each subset of characters and produced an ordination using the same methods as outlined above. An initial morphospace produced for branching anatomy and appendages was heavily distorted by the lack of homology between euphyllophytes and other land plants and so the space was recreated solely for euphyllophytes.

### The evolution of complexity

To quantify complexity, as opposed to disparity, we edited the character matrix such that, where possible, each character instead reflected the presence or absence of a trait (data available online). Characters that could not feasibly be edited into this format were excluded. Each of the remaining characters was coded with absence as ‘0’ and presence as ‘1’, allowing a sum of the total number of present characters to be calculated. As previously, we reconstructed each of the characters along the phylogeny, estimating the complexity at each internal node along the tree. As a potential means for explaining variation in complexity, we summed the total number of polyploidy events undergone by each species in the tree (data available online). We compared the number of ploidy events with complexity using a Spearman’s correlation.

To ensure that the deletion of certain characters did not affect our results, we repeated the analysis with the original matrix, instead summing the proportion of applicable characters as a proxy for complexity.

### Reporting summary

Further information on research design is available in the Nature Portfolio Reporting Summary linked to this article.

### Supplementary information

Reporting Summary

## Figures and Tables

**Extended Data Fig. 6:**
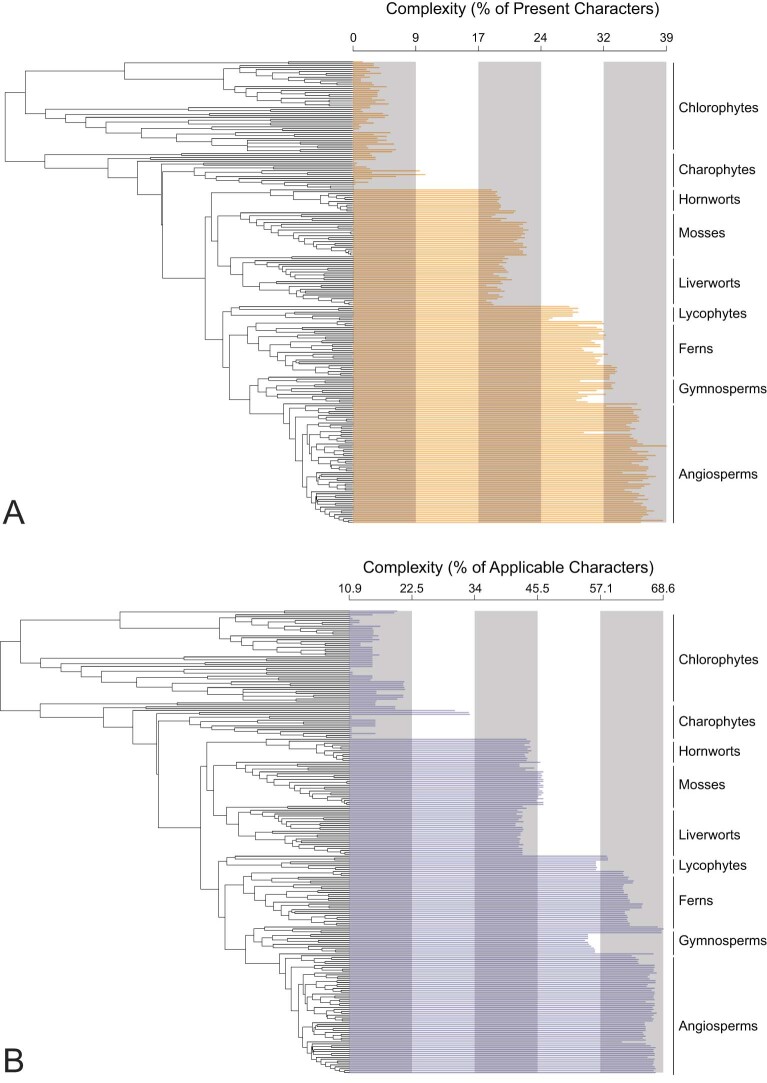
Phenotypic complexity of the Plant Kingdom. Comparison of object complexity based on A. the number of positively coded characters for each taxon and B. the number of applicable characters for each taxon included in the analysis.

**Extended Data Fig. 7:**
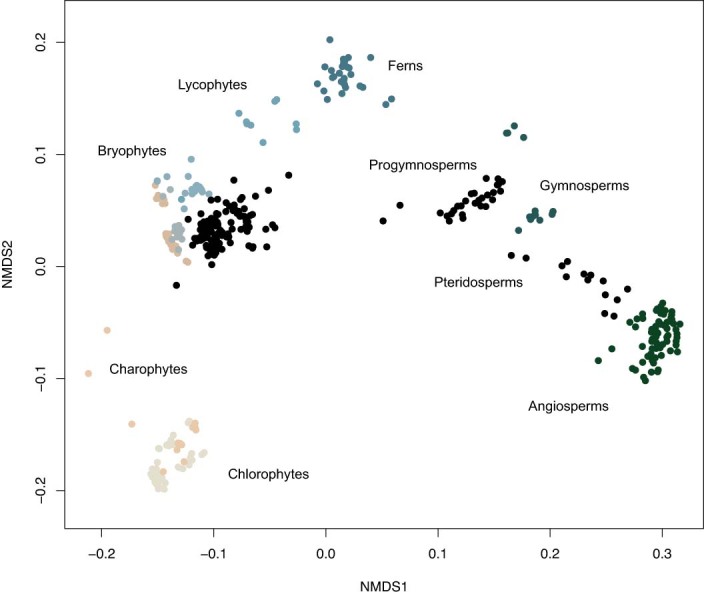
A morphospace including extant (coloured dots) and fossil (black dots) species in which missing data among the fossil species have been scored an unknown (‘?’). Note the clustering of fossil taxa on NMDS 2 and the grouping of stem-tracheophytes, -lycophytes and -euphyllophytes with extant bryophytes.

**Extended Data Fig. 8:**
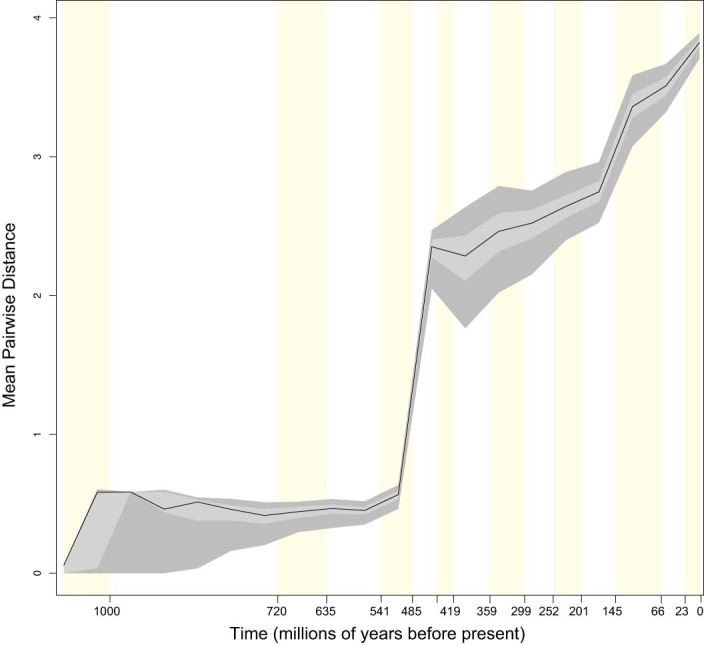
Cumulative phenotypic disparity (sum of variances) through time. This plot differs from Fig. 4b, in that the divergence of crown-angiosperms is constrained to the early Cretaceous.

## Data Availability

The data used in our analyses are publicly available from the Bristol Research Data Facility: 10.5523/bris.1j3vex0yas0rz2ku42prh7evx9.

## Code availability

The code used in our analyses are publicly available from the Bristol Research Data Facility: 10.5523/bris.1j3vex0yas0rz2ku42prh7evx9.
