# Supplementary material for: Evolution of phenotypic disparity in the plant kingdom
Source: Nat Plants. 2023 Sep 4;9(10):1618–26. doi: 10.1038/s41477-023-01513-x (PMC10581900; doi:10.1038/s41477-023-01513-x)
Supplement: Supplementary file 1 — Reporting Summary [file 41477_2023_1513_MOESM1_ESM.pdf]

nature portfolio

Corresponding author(s): Philip Donoghue

Last updated by author(s): Aug 3, 2023

Reporting Summary

Nature Portfolio wishes to improve the reproducibility of the work that we publish. This form provides structure for consistency and transparency in reporting. For further information on Nature Portfolio policies, see our Editorial Policies and the Editorial Policy Checklist.

Statistics

For all statistical analyses, confirm that the following items are present in the figure legend, table legend, main text, or Methods section.

n/a	Confirmed	
☐	☒	The exact sample size (*n*) for each experimental group/condition, given as a discrete number and unit of measurement
☐	☒	A statement on whether measurements were taken from distinct samples or whether the same sample was measured repeatedly
☐	☒	The statistical test(s) used AND whether they are one- or two-sided<br>*Only common tests should be described solely by name; describe more complex techniques in the Methods section.*
☐	☒	A description of all covariates tested
☒	☐	A description of any assumptions or corrections, such as tests of normality and adjustment for multiple comparisons
☐	☒	A full description of the statistical parameters including central tendency (e.g. means) or other basic estimates (e.g. regression coefficient) AND variation (e.g. standard deviation) or associated estimates of uncertainty (e.g. confidence intervals)
☐	☒	For null hypothesis testing, the test statistic (e.g. *F*, *t*, *r*) with confidence intervals, effect sizes, degrees of freedom and *P* value noted<br>*Give P values as exact values whenever suitable.*
☐	☒	For Bayesian analysis, information on the choice of priors and Markov chain Monte Carlo settings
☒	☐	For hierarchical and complex designs, identification of the appropriate level for tests and full reporting of outcomes
☐	☒	Estimates of effect sizes (e.g. Cohen's *d*, Pearson's *r*), indicating how they were calculated

Our web collection on statistics for biologists contains articles on many of the points above.

Software and code

Policy information about availability of computer code

Data collection	No software was used for data collection.
Data analysis	dispRity - disparity analyses. Guillerme, T., and Poisot, T., 2018, dispRity: A modular R package for measuring disparity: Methods in Ecology and Evolution, v. 9, p. 1755-1763.<br>MrBayes - Bayesian phylogenetic analyses. Ronquist, F., Teslenko, M., van der Mark, P., Ayres, D. L., Darling, A., Hohna, S., Larget, B., Liu, L., Suchard, M. A., and Huelsenbeck, J. P., 2012, MrBayes 3.2: efficient Bayesian phylogenetic inference and model choice across a large model space: Systematic Biology, v. 61, no. 3, p. 539-542.<br>phytools - stochastic character state mapping. Revell, L. J., 2012, phytools: An R package for phylogenetic comparative biology (and other things): Methods in Ecology and Evolution, v. 3, p. 217-223.<br>R - statistical analyses. Team, R. C., 2021, R: A language and environment for statistical computing: Vienna, Austria, . R Foundation for Statistical Computing. Version 4.0.<br>vegan - disparity analyses. Oksanen, J., Blanchet, F. G., Kindt, R., Legendre, P., Minchin, P. R., O'Hara, R. B., Simpson, G. L., Solymos, P., Stevens, M. H. H., and Wagner, H., 2013, vegan: Community Ecology Package. R package version 2.0-7: Available at: http://CRAN.Rproject.org/package=vegan.

For manuscripts utilizing custom algorithms or software that are central to the research but not yet described in published literature, software must be made available to editors and reviewers. We strongly encourage code deposition in a community repository (e.g. GitHub). See the Nature Portfolio guidelines for submitting code & software for further information.

Data

Policy information about availability of data

All manuscripts must include a data availability statement. This statement should provide the following information, where applicable:

- Accession codes, unique identifiers, or web links for publicly available datasets
- A description of any restrictions on data availability
- For clinical datasets or third party data, please ensure that the statement adheres to our policy

The data used in our analyses are publicly available from the Bristol Research Data Facility: DOI: DOI:10.5523/bris.1j3vex0yas0rz2ku42prh7evx9

Research involving human participants, their data, or biological material

Policy information about studies with human participants or human data. See also policy information about sex, gender (identity/presentation), and sexual orientation and race, ethnicity and racism.

Reporting on sex and gender	Not applicable.
Reporting on race, ethnicity, or other socially relevant groupings	Not applicable.
Population characteristics	Not applicable.
Recruitment	Not applicable.
Ethics oversight	Not applicable.

Note that full information on the approval of the study protocol must also be provided in the manuscript.

Field-specific reporting

Please select the one below that is the best fit for your research. If you are not sure, read the appropriate sections before making your selection.

☐ Life sciences ☐ Behavioural & social sciences ☒ Ecological, evolutionary & environmental sciences

For a reference copy of the document with all sections, see nature.com/documents/nr-reporting-summary-flat.pdf

Ecological, evolutionary & environmental sciences study design

All studies must disclose on these points even when the disclosure is negative.

Study description	Phenotypic disparity analysis of the plant kingdom
Research sample	Collated, reviewed and revised data from published phylogenetic studies, supplemented by direct observations.
Sampling strategy	Capture plant phenotype across the phylogenetic breadth of the plant kingdom
Data collection	Collated, reviewed and revised data from published phylogenetic studies, supplemented by direct observations.
Timing and spatial scale	2012-2023
Data exclusions	Not applicable.
Reproducibility	The sources are cited.
Randomization	Not applicable.
Blinding	Not applicable.

Did the study involve field work? ☐ Yes ☒ No

Reporting for specific materials, systems and methods

We require information from authors about some types of materials, experimental systems and methods used in many studies. Here, indicate whether each material, system or method listed is relevant to your study. If you are not sure if a list item applies to your research, read the appropriate section before selecting a response.

Materials & experimental systems

n/a	Involved in the study	
☒	☐	Antibodies
☒	☐	Eukaryotic cell lines
☒	☐	Palaeontology and archaeology
☒	☐	Animals and other organisms
☒	☐	Clinical data
☒	☐	Dual use research of concern
☒	☐	Plants

Methods

n/a	Involved in the study	
☒	☐	ChIP-seq
☒	☐	Flow cytometry
☒	☐	MRI-based neuroimaging
